# Clinical outcomes of asymptomatic low-grade esophagitis: results from a multicenter Chinese cohort

**DOI:** 10.1093/gastro/goac057

**Published:** 2022-10-13

**Authors:** Songfeng Chen, Xuelian Xiang, Xiaohao Zhang, Qianjun Zhuang, Niandi Tan, Xun Hou, Mengyu Zhang, Junnan Hu, Chaofan Duan, Yi Cui, Jinhui Wang, Xiangbin Xing, Nina Zhang, Yinglian Xiao

**Affiliations:** Department of Gastroenterology, The First Affiliated Hospital of Sun Yat-sen University, Guangzhou, Guangdong, P. R. China; Department of Gastroenterology, Union Hospital, Tongji Medical College, Huazhong University of Science and Technology, Wuhan, Hubei, P. R. China; Department of Gastroenterology, Union Hospital, Tongji Medical College, Huazhong University of Science and Technology, Wuhan, Hubei, P. R. China; Department of Gastroenterology, The First Affiliated Hospital of Sun Yat-sen University, Guangzhou, Guangdong, P. R. China; Department of Gastroenterology, The First Affiliated Hospital of Sun Yat-sen University, Guangzhou, Guangdong, P. R. China; Gastrointestinal Surgery Center, The First Affiliated Hospital of Sun Yat-sen University, Guangzhou, Guangdong, P. R. China; Department of Gastroenterology, The First Affiliated Hospital of Sun Yat-sen University, Guangzhou, Guangdong, P. R. China; Department of Gastroenterology, The First Affiliated Hospital of Sun Yat-sen University, Guangzhou, Guangdong, P. R. China; Department of Gastroenterology, Union Hospital, Tongji Medical College, Huazhong University of Science and Technology, Wuhan, Hubei, P. R. China; Department of Gastroenterology, The First Affiliated Hospital of Sun Yat-sen University, Guangzhou, Guangdong, P. R. China; Department of Gastroenterology, The First Affiliated Hospital of Sun Yat-sen University, Guangzhou, Guangdong, P. R. China; Department of Gastroenterology, The First Affiliated Hospital of Sun Yat-sen University, Guangzhou, Guangdong, P. R. China; Department of Gastroenterology, The Affiliated Drum Tower Hospital of Nanjing University Medical School, Nanjing, Jiangsu, P. R. China; Department of Gastroenterology, The First Affiliated Hospital of Sun Yat-sen University, Guangzhou, Guangdong, P. R. China

**Keywords:** esophagitis, asymptomatic, proton-pump inhibitor, symptom, mucosal

## Abstract

**Background:**

Asymptomatic low-grade (Los Angeles Classification Grades A and B) esophagitis is common in clinical practice with unclear clinical outcomes. This study aimed to explore the clinical outcomes of asymptomatic low-grade esophagitis.

**Methods:**

This was a multicenter cohort study conducted by three academic hospitals in China. Asymptomatic low-grade esophagitis patients between January 2015 and December 2019 were included. Mucosal healing condition 1 year after initial diagnosis, symptom outcomes, and proton-pump inhibitor (PPI) use within 1 year after initial diagnosis were studied and compared.

**Results:**

A total of 248 asymptomatic low-grade esophagitis patients were included. Esophagitis disappeared in 76.2% of patients 1 year after initial diagnosis. In terms of symptom outcomes, 89.9% of patients did not present gastroesophageal reflux disease (GERD) symptoms within 1 year after initial diagnosis. No significant difference was found in the proportion of patients who presented GERD symptoms and in the proportion of patients with persistent esophagitis between the PPI group and the non-PPI group (all *P *>* *0.05). Patients with initial Grade B esophagitis were more likely to present follow-up GERD symptoms (16.0% vs 7.5%, *P *=* *0.041) and had more severe follow-up esophagitis than those with Grade A (*P *<* *0.001). Patients with follow-up GERD symptoms were more likely to have persistent esophagitis than those without.

**Conclusions:**

This study demonstrated that asymptomatic low-grade esophagitis had relatively benign clinical outcomes. Patients with initial Grade B esophagitis and patients with follow-up GERD symptoms were more likely to be those who are in genuine need of further follow-up and treatments.

## Introduction

Gastroesophageal reflux disease (GERD) is a common chronic disease caused by the reflux of gastroduodenal contents entering into the esophagus or mouth. It can be divided into non-erosive gastroesophageal reflux disease (combined with abnormal ambulatory reflux monitoring), reflux esophagitis (RE), and Barrett's esophagus (BE) according to the endoscopic manifestations [1]. Epidemiological studies showed that the prevalence of GERD is up to 8%–33% worldwide, covering all age groups and both genders [2]. GERD not only affects patients’ quality of life and brings huge economic burden to social medical resources [3], but also plays an important role in the occurrence of esophageal cancer [4].

GERD is heterogeneous in the clinical scenario since the symptom does not correlate well with the mucosal injury, let alone the acid exposure during the ambulatory esophageal monitoring. This increases the difficulty of diagnosing GERD. In order to clarify the diagnosis of GERD, the Lyon consensus has stated that high-grade esophagitis (Los Angeles [LA] Classification Grades C or D), BE, or peptic stricture are considered to be confirmatory endoscopic evidence of GERD [5]. Previous studies have shown that BE might develop if esophagitis remained untreated and a higher grade of esophagitis might be more possible to develop into BE, and even into esophageal adenocarcinoma [6, 7]. Therefore, monitoring the endoscopic appearance among patients with severe esophagitis seems necessary. Furthermore, proton-pump inhibitor (PPI) use is recommended as the preliminary diagnostic test for patients with typical reflux symptoms (regardless of whether they have esophagitis or not) [8]. However, in clinical practice, there is still another kind of patient—asymptomatic low-grade esophagitis. With regard to this type of patient, there are limited studies and standardized management suggestion is lacking. Indeed, asymptomatic low-grade esophagitis can be found in ∼6.3%–12.0% of the healthy population [9–13]. It remains unclear whether further evaluation to look for the additional evidence of GERD and long-term follow-up, as well as the standardized acid inhibitory treatment, were necessary due to the vague clinical outcome of such asymptomatic low-grade esophagitis.

Clarifying the clinical outcomes of asymptomatic low-grade esophagitis and identifying patients who are in genuine need of follow-up examinations and treatments would help to guide management and avoid unnecessary use of acid suppressors. The aim of this study was to explore the clinical outcomes of asymptomatic low-grade esophagitis.

## Methods

### Study subjects

This was a multicenter retrospective cohort study conducted by three academic hospitals in China (The First Affiliated Hospital of Sun Yat-sen University, Guangzhou, Guangdong; The Affiliated Drum Tower Hospital of Nanjing University Medical School, Nanjing, Jiangsu; Union Hospital, Tongji Medical College, Huazhong University of Science and Technology, Wuhan, Hubei). Patients who met all the following criteria were included: (i) were 18–75 years old; (ii) had received endoscopy evaluation for routine physical examination between January 2015 and December 2019 without gastrointestinal (GI) symptoms; (iii) had been diagnosed with low-grade esophagitis under endoscopy evaluation (LA Grades A or B) [14]; (iv) had received endoscopic and symptom review 1 year (±3 months) after initial diagnosis. Patients who met any of the following criteria were excluded: (i) had any upper GI symptoms 3 months prior to the initial diagnosis; (ii) had gastroesophageal surgery or tumor history; (iii) had rheumatic immune diseases; (iv) had received PPI, prokinetic drugs, or antacids 2 weeks prior to the initial diagnosis; (v) had any other organic diseases under endoscopy evaluation; (vi) had incomplete demographic, symptom, or medication information. This study was approved by the Ethical Review Board of the three academic hospitals (IRB no. 2021275; IRB no. 2021–425-01; IRB no. 20210662) and was performed following the concept of the Declaration of Helsinki.

### Endoscopy evaluation

As is routine in these academic hospitals, demographic data, symptom information, and medication use were recorded each time before the endoscopy examination.

Endoscopic images of patients who underwent endoscopy evaluation for routine physical examination without GI symptoms between January 2015 and December 2019 were extracted. The endoscopic images were reviewed by two independent experienced gastroenterologists. The presence and severity of esophagitis were evaluated based on LA Classification. LA Classification is as follows: (i) Grade A represents one or more erosion(s) of <5 mm that does not extend between the tops of two mucosal folds; (ii) Grade B corresponds to one or more erosion(s) with length of >5 mm that does not extend between the tops of two mucosal folds; (iii) Grade C is one or more erosion(s) that is continuous between the tops of two or more mucosal folds but that involves <75% of the circumference; (iv) Grade D is one or more erosion(s) that involves ≥75% of the esophageal circumference [14]. If the judgments of the two gastroenterologists are inconsistent, a third senior professor will make the final judgment. If these patients fulfilled the inclusion criteria and did not meet any exclusion criteria, their follow-up endoscopic images would be also extracted and assessed using LA Classification.

### Assessment of index

The primary outcome of the study was mucosal healing condition, i.e. the presence of persistent esophagitis. The secondary outcomes were the presence of follow-up GERD symptoms.

Persistent esophagitis was defined as any grade of RE discovered by follow-up endoscopy examination 1 year after initial diagnosis. The presence of follow-up GERD symptoms was defined as new onset heartburn and/or regurgitation occurring at least three times per week and persisting for >3 months within 1 year after the initial diagnosis [15]. PPI use was defined as the use of any PPI for >8 weeks [8].

### Statistical analysis

Frequency (percentage) and chi-square test was used for categorical variables. For continuous variables, the Kolmogorov–Smirnov test was used to identify whether the variables were normally distributed or not. If the variables were normally distributed, mean* *±* *standard deviation (SD) and *t*-test were used, otherwise median (25th, 75th) and Kruskal–Wallis test were used. Analyses were conducted using IBM SPSS Statistics 22 (IBM, Armonk, New York, USA). The significant difference was set at *P *<* *0.05.

## Results

A total of 10,788 asymptomatic patients underwent routine endoscopes between 2015 and 2019 in the three academic hospitals. Low-grade esophagitis was found in 841 patients (accounting for 7.8% of asymptomatic patients who underwent endoscopes), 542 (64.4%) of whom were identified as Grade A esophagitis patients and 299 (35.6%) Grade B. Among these 841 asymptomatic low-grade esophagitis patients, 593 patients were excluded due to the lack of complete follow-up data (PPI use information, symptom outcomes, or mucosal healing conditions). Therefore, 248 patients with complete follow-up information were finally included in this study.

### Overall outcome

Esophagitis disappeared in 76.2% (189 of 248) of asymptomatic low-grade patients (including those who received PPI treatment and those who did not) 1 year after initial diagnosis. In terms of symptom outcomes, 89.9% (223 of 248) of asymptomatic low-grade esophagitis patients (including those who received PPI treatment and those who did not) did not present GERD symptoms within 1 year after initial diagnosis. Only one case (initial esophagitis: Grade A; received PPI treatment for 12 weeks) of asymptomatic low-grade esophagitis upgraded (follow-up esophagitis: Grade C) during the study period. No complication (esophageal stricture, BE) of GERD happened in the current study. Therefore, the overall clinical outcome of asymptomatic low-grade esophagitis was relatively benign.

### Comparison of initial esophagitis severity

Patients were divided into Group A (esophagitis with LA-A grade) (*n *=* *173) and Group B (esophagitis with LA-B grade) (*n *=* *75) according to their initial esophagitis severity. There were more male patients, as well as more patients who received PPI treatment in Group B compared with that in Group A (88.0% vs 76.3%, *P *=* *0.035; and 45.3% vs 30.0%, *P *=* *0.020, respectively; Table 1). Patients in Group B were more likely to present GERD symptoms within 1 year after initial diagnosis (16.0% vs 7.5%, *P *=* *0.041). Patients in Group B had more severe follow-up esophagitis than those in Group A (*P *<* *0.001).

**Table 1.** Comparison of initial esophagitis severity

Characteristic	Grade A	Grade B	*P*-value
	(*n *=* *173)	(*n *=* *75)	
Male, *n* (%)	132 (76.3)	66 (88.0)	0.035
Age, mean ± SD, years	50.6 ± 12.2	48.5 ± 10.4	0.191
Body mass index, mean ± SD, kg/m^2^	23.7 ± 3.1	23.5 ± 3.0	0.559
Received PPI treatment, *n* (%)	52 (30.0)	34 (45.3)	0.020
Follow-up GERD symptoms, *n* (%)	13 (7.5)	12 (16.0)	0.041
Follow-up esophagitis severity, *n* (%)			<0.001
None	133 (76.9)	56 (74.7)	
Grade A	39 (22.5)	6 (8.0)	
Grade B	0 (0)	13 (17.3)	
Grade C	1 (0.6)	0 (0)	
Grade D	0 (0)	0 (0)	

Grade A, Los Angeles Classification Grade A esophagitis; Grade B, Los Angeles Classification Grade B esophagitis; SD, standard deviation; PPI, proton-pump inhibitor; GERD, gastroesophageal reflux disease.

### Comparison of PPI treatment conditions

Eighty-six patients received PPI treatment, while the other 162 patients did not receive PPI treatment within 1 year after initial diagnosis. Initial esophagitis was found to be more severe in the PPI group (*P *=* *0.020, Table 2). No significant difference was found in gender, age, and body mass index (BMI). For the proportion of patients presented GERD symptoms, there was no significant difference between the PPI group and the non-PPI group (low-grade: 12.7% vs 8.7%, *P *=* *0.302; Grade A: 9.7% vs 6.7%; *P *=* *0.492; Grade B: 17.7% vs 14.7%, *P *=* *0.723). As for the proportion of patients with persistent esophagitis, no significant difference was found between the PPI group and the non-PPI group regardless of the initial esophagitis severity either (low-grade: 30.2% vs 20.4%, *P *=* *0.083; Grade A: 28.8% vs 20.7%, *P *=* *0.242; Grade B: 32.3% vs 19.6%, *P *=* *0.204). Thus, indiscriminate administration of PPI therapy in all asymptomatic low-grade esophagitis patients might not be appropriate.

**Table 2.** Comparison of PPI treatment conditions

Characteristic	PPI group	Non-PPI group	*P*-value
	(*n *=* *86)	(*n *=* *162)	
Male, *n* (%)	68 (79.1)	130 (80.2)	0.826
Age, mean ± SD, years	48.4 ± 11.6	50.8 ± 11.7	0.118
Body mass index, mean ± SD, kg/m^2^	23.7 ± 3.3	23.6 ± 3.0	0.859
Initial esophagitis severity, *n* (%)			0.020
Grade A	52 (60.5)	121 (74.7)	
Grade B	34 (39.5)	41 (25.3)	

PPI, proton-pump inhibitor; SD, standard deviation.

### Comparison of follow-up evaluation

In order to identify clinically significant patients, patients were further divided into different groups according to their follow-up clinical outcomes. Patients were divided into the GERD symptom group (*n *=* *25) and the non-GERD symptom group (*n *=* *223) according to whether they presented GERD symptoms within 1 year after the initial diagnosis. No significant difference was found in age, gender, and BMI (Table 3). Patients with GERD symptoms had a higher grade of initial esophagitis and follow-up esophagitis (*P < *0.001; Table 3). Patients were also divided into the persistent esophagitis group (*n *=* *59) and the non-persistent esophagitis group (*n *=* *189) according to their follow-up endoscopic outcomes. It showed that patients with persistent esophagitis were more likely to present GERD symptoms within 1 year after the initial diagnosis (27.1% vs 4.8%, *P < *0.001; Table 4). These results suggested that patients with initial Grade B esophagitis and patients with follow-up GERD symptoms might be those who are in genuine need of further follow-up and treatments. Further examinations and treatments should be considered in these patients to prevent the occurrence of GERD and long-term esophageal injury.

**Table 3.** Comparison of follow-up GERD symptoms

Characteristic	GERD symptom group	Non-GERD symptom group	*P*-value
	(*n *=* *25)	(*n *=* *223)	
Male, *n* (%)	21 (84.0)	177 (79.4)	0.584
Age, mean ± SD, years	47.0 ± 10.1	50.3 ± 11.9	0.180
Body mass index, mean ± SD, kg/m^2^	23.7 ± 2.7	23.7 ± 3.1	0.961
Initial esophagitis severity, *n* (%)			0.041
Grade A	13 (52.0)	160 (71.7)	
Grade B	12 (48.0)	63 (28.3)	
Received PPI treatment, *n* (%)	11 (44.0)	75 (33.6)	0.302
Follow-up esophagitis severity, *n* (%)			<0.001
0	9 (36.0)	180 (80.8)	
A	11 (44.0)	34 (15.2)	
B	4 (16.0)	9 (4.0)	
C	1 (4.0)	0 (0.0)	

GERD, gastroesophageal reflux disease; SD, standard deviation; PPI, proton-pump inhibitor.

**Table 4.** Comparison of follow-up esophagitis conditions

Characteristic	Persistent esophagitis group	Non-persistent esophagitis group	*P*-value
	(*n *=* *59)	(*n *=* *189)	
Male, *n* (%)	47 (79.7)	151 (79.9)	0.969
Age, mean ± SD, years	49.6 ± 12.5	50.1 ± 11.5	0.801
Body mass index, mean ± SD, kg/m^2^	22.9 ± 3.4	23.9 ± 2.9	0.064
Initial esophagitis severity, *n* (%)			0.707
Grade A	40 (67.8)	133 (70.4)	
Grade B	19 (32.2)	56 (29.6)	
Received PPI treatment, *n* (%)	26 (44.1)	60 (31.7)	0.083
Follow-up GERD symptoms, *n* (%)	16 (27.1)	9 (4.8)	<0.001

PPI, proton-pump inhibitor; GERD, gastroesophageal reflux disease; SD, standard deviation.

## Discussion

GERD is a complex disease with heterogeneous symptom profiles and endoscopic manifestations. Although patients with typical symptoms or high-grade esophagitis can be diagnosed with GERD, asymptomatic low-grade esophagitis patients are also often seen in clinical practice. However, little attention has been attached to asymptomatic low-grade esophagitis so far. In this study, asymptomatic low-grade esophagitis patients were found to have benign clinical outcomes. Most of the esophagitis disappeared 1 year after the initial diagnosis. Most patients did not present GERD symptoms within 1 year after initial diagnosis. Receiving PPI treatment could neither improve the mucosal healing condition nor prevent the GERD symptoms. Patients with initial asymptomatic Grade B esophagitis were more likely to present follow-up GERD symptoms than those with Grade A. Patients with follow-up GERD symptoms were more likely to have persistent esophagitis.

LA Classification is the most widely used esophagitis grading system in clinical practice. It is associated with symptom severity, acid exposure, and esophageal motility abnormalities. Therefore, it is believed that LA Classification can be used to indicate the severity of GERD disease and predict the therapeutic effect and clinical prognosis [14, 16]. The diagnostic values of different grades of esophagitis were evaluated by the Lyon consensus. While high-grade esophagitis was considered as confirmatory evidence for GERD, both Grade A and Grade B esophagitis were considered to be borderline or inconclusive evidence [5]. American College of Gastroenterology (ACG) clinical guidelines also pointed out that both Grade A and Grade B esophagitis were not sufficient for a definitive diagnosis of GERD alone [17]. In this study, patients with initial Grade B esophagitis were more likely to present follow-up GERD symptoms and had more severe follow-up esophagitis than those with Grade A. This might be caused by the highly subjective diagnosis of Grade A esophagitis (as only erosion of esophagus of <5 mm is classified into Grade A). Therefore, it might be inappropriate to position Grade A and Grade B esophagitis as the same level of evidence for GERD. Further studies focusing on the different diagnostic values between Grade A and Grade B esophagitis are still needed.

So far, limited studies have focused on esophagitis in asymptomatic people. Akdamar *et al.* [11] prospectively included 355 healthy, asymptomatic, male volunteers. Esophagitis was found in 8.5% of healthy volunteers. A study by Zagari *et al.* [10] found that esophagitis was presented in 7.0% of asymptomatic patients. Mild esophagitis accounted for 90.0% of asymptomatic esophagitis [10]. A Japanese study also found that 11.2% of healthy volunteers have esophagitis, among whom 96.4% of them were low-grade. Some studies have also attempted to identify risk factors for asymptomatic low-grade esophagitis. However, the results of these studies have been inconsistent. In a Korean study, Jung *et al.* [18] found that non-smoking habit and lower BMI were associated with asymptomatic RE. However, in a Chinese Taiwan study, Wang *et al.* [13] included 70 asymptomatic esophagitis patients and found that male gender and higher BMI were the risk factors for asymptomatic esophagitis. Younger age was also found to be related to asymptomatic esophagitis by a recent study in Nepal [12]. Heterogeneity in the results of these studies may have been caused by the small sample sizes and retrospective design of these studies. Furthermore, none of the previous studies followed up these patients to explore their clinical outcomes. So whether we should provide acid suppression for these asymptomatic low-grade esophagitis patients remained unclear, subsequently leading to the various management strategies for these patients. To our knowledge, our current study was the first to explore the clinical significance of asymptomatic low-grade esophagitis. Considering the benign outcomes of these patients, administering acid suppression to these patients should be cautious. However, we did find that patients with initial Grade B esophagitis were more likely to present follow-up GERD symptoms and had more severe follow-up esophagitis. Furthermore, patients with follow-up GERD symptoms were more likely to have persistent esophagitis. Thus, these patients might be those who really need follow-up examinations and treatments.

The pathogenesis of asymptomatic low-grade esophagitis remained uncertain. Although gastroesophageal reflux is the most common cause of esophagitis, esophagitis can also be caused by radiation, infections, local injury caused by medications or food, immune-related disorder, etc. [19]. Not all the causes are acid-related. Some types of esophagitis are temporary lesions of the esophagus and may not require medical treatment. This was confirmed through the current study in which asymptomatic low-grade esophagitis demonstrated a relatively good prognosis. Besides, the clinical outcomes between the PPI group and the non-PPI group were comparable. Further studies focusing on the differences in pathogenesis between symptomatic esophagitis and asymptomatic esophagitis are needed.

This study also had some limitations. First, it was a retrospective study. Thus, treatments (e.g. the type of PPI, the start time of the treatment and the treatment duration, the treatment compliance, and the use of drugs other than PPI) may vary between patients. Endoscopic capture of images at the gastroesophageal junction was also not standardized due to the retrospective design of this study. Prospective clinical studies are still needed to further confirm our findings. Second, symptom evaluation was primarily subjective and no standardized patient-reported outcome measures were used. Third, patients were followed up for only 1 year, which might not be long enough for patients to develop symptoms (especially when the requirement for symptom duration in this study was 3 months) or complications. It is unclear whether the benefits of PPI will be seen over longer follow-up periods. Fourth, while analysing follow-up symptom outcomes, only typical GERD symptoms were analysed in this study. Finally, no motility and reflux monitoring data were available. These patients were asymptomatic, which somehow resulted in the low willingness to conduct high-resolution manometry and reflux monitoring.

In conclusion, this multicenter cohort study demonstrated that asymptomatic low-grade esophagitis had relatively benign clinical outcomes. Indiscriminate administration of acid-suppressive agents to all patients with asymptomatic low-grade esophagitis does not help improve the clinical outcome of these patients. Patients with initial Grade B esophagitis and patients with follow-up GERD symptoms might be the ones who are in genuine need of further follow-up and treatments.

## Authors’ Contributions

S.C. and Xuelian Xiang were responsible for the acquisition, analysis, and interpretation of data, and drafting of the manuscript; X.Z., Q.Z., N.T., X.H., M.Z., J.H., C.D., Y.C., J.W., and Xiangbin Xing were responsible for interpretation of data; N.Z. and Y.X. were responsible for conception and design of the study, data analysis, and finalization of the manuscript. All authors have read and approved the final version of the manuscript.

## Funding

This work was supported by the National Natural Science Foundation of China [grant numbers 81770544 and 81970479].

## Conflict of Interest

There is no conflict of interest in this study.
